# Tim-3 blocking rescue macrophage and T cell function against *Mycobacterium tuberculosis* infection in HIV+ patients

**DOI:** 10.7448/IAS.18.1.20078

**Published:** 2015-10-19

**Authors:** Isabel Sada-Ovalle, Ranferi Ocaña-Guzman, Santiago Pérez-Patrigeón, Leslie Chávez-Galán, Juan Sierra-Madero, Luis Torre-Bouscoulet, Marylyn M. Addo

**Affiliations:** 1 Laboratorio de Inmunología IntegrativaInstituto Nacional de Enfermedades Respiratorias Ismael Cosío Villegas, Distrito Federal, México; 2 Departamento de Infectología del Instituto Nacional de Ciencias Médicas y Nutrición “Salvador Zubirán”, Distrito Federal, México; 3 Department of Internal Medicine, University Medical Center Hamburg-Eppendorf, Hamburg, Germany; 4 German Center for Infection Research, Partner Site Standort Hamburg-Lübeck-Borstel

**Keywords:** HIV, tuberculosis, Tim-3, galectin 9, PD-1

## Abstract

**Introduction:**

T cell immunoglobulin and mucin domain (Tim) 3 and programmed death 1 (PD-1) are co-inhibitory receptors involved in the so-called T cell exhaustion, and in vivo blockade of these molecules restores T cell dysfunction. High expression of Tim-3 and PD-1 is induced after chronic antigen-specific stimulation of T cells during HIV infection. We have previously demonstrated that the interaction of Tim-3 with its ligand galectin-9 induces macrophage activation and killing of Mycobacterium tuberculosis. Our aim in this study was to analyze the Tim-3 expression profile before and after six months of antiretroviral therapy and the impact of Tim-3 and PD-1 blocking on immunity against M. tuberculosis.

**Materials and methods:**

HIV+ patients naïve to anti-retroviral therapy (ART) were followed up for six months. Peripheral immune-cell phenotype (CD38/HLA-DR/galectin-9/Tim-3 and PD-1) was assessed by flow cytometry. Supernatants were analyzed with a multiplex cytokine detection system (human Th1/Th2 cytokine Cytometric Bead Array) by flow cytometry. Control of bacterial growth was evaluated by using an *in vitro* experimental model in which virulent *M. tuberculosis*-infected macrophages were cultured with T cells in the presence or absence of Tim-3 and PD-1 blocking antibodies. Interleukin-1 beta treatment of infected macrophages was evaluated by enumerating colony-forming units.

**Results:**

We showed that HIV+ patients had an increased expression of Tim-3 in T cells and were able to control bacterial growth before ART administration. By blocking Tim-3 and PD-1, macrophages and T cells recovered their functionality and had a higher ability to control bacterial growth; this result was partially dependent on the restitution of cytokine production.

**Conclusions:**

In this study, we demonstrated that increased Tim-3 expression can limit the ability of the immune system to control the infection of intracellular bacteria such as *M. tuberculosis*. The use of ART and the *in vitro* manipulation of the Tim-3 and PD-1 molecules restored the functionality of T cells and macrophages to restrict bacterial growth. Our results provide a novel immune strategy that may be implemented in the near future in order to improve the immune responses in HIV+ patients.

## Introduction

Pulmonary tuberculosis (TB) is one of the most important causes of death from infectious disease around the world. It is the leading cause of opportunistic infection and mortality among people living with HIV infection. According to the World Health Organization, HIV+ patients have an estimated 20 to 30 times greater risk of developing active pulmonary TB than HIV people [1, 2]. HIV/AIDS is a disease characterized by the progressive loss of CD4 T cells that leads to severe immunodeficiency. When CD4 T cell levels fall below 200 cells/mm^3^, the risk of infection by opportunistic pathogens such as *Mycobacterium tuberculosis* (M.tb) increases greatly. Thus, one of the main goals of antiretroviral therapy (ART) is to prevent the development of opportunistic infections in order to reduce the mortality of infected patients [3].

T cell immunoglobulin- and mucin-domain-containing molecule 3 (Tim-3) is a type I transmembrane protein expressed in T cells, monocytes, macrophages and dendritic cells (DCs) [4]. Interaction of Tim-3 with its ligand, galectin-9 (Gal9), expressed by myeloid cells (monocytes, DCs and macrophages), modulates immune responses by promoting the death of CD4+ Th1 cells through a mechanism involving Th1 calcium fluxes [5]. Together with the negative regulator programmed death-1 (PD-1), Tim-3 expression is associated with a dysfunctional T cell phenotype in many viral infections such as HIV and hepatitis C and B [6, 7]. Several reports have shown that specific blocking of Tim-3 and PD-1 signaling pathways improved T cell responses and viral control in chronically infected patients [8, 9].

We have demonstrated that Tim-3/Gal9 interaction induces an activation program in M.tb-infected macrophages, resulting in IL-1β secretion and pathogen clearance [10]. Those findings suggested that this interaction acts as a bidirectional pathway which regulates both the innate and adaptive arms of the immune system. While this interaction might have evolved as a means of limiting tissue inflammation caused by activated Th1 cells, it can also stimulate innate immunity and thus inhibit the growth of intracellular pathogens [10].

The goal of this study was to assess the phenotypic and functional traits of CD4 + Tim-3+ and CD8 + Tim-3+ T cells before and during the first six months of ART in HIV+ patients using an *in vitro* model of M.tb infection.

## Methods

### Study population

This study was conducted at the Instituto Nacional de Enfermedades Respiratorias (INER) and the Instituto Nacional de Ciencias Médicas y Nutrición Salvador Zubirán (INCMNSZ) in Mexico City. Twenty ≥18-year-old HIV patients (HIV+ patients) naïve to ART were included and followed up for six months. The control group included 20 healthy blood donors. A PPD skin test was performed using the Mantoux method. A positive skin test in HIV+ patients was defined as an induration area ≥5 mm in diameter, whereas in the control group the measure was ≥10 mm. HIV+ patients did not have history of pulmonary TB or symptoms of pulmonary diseases (TB), and the PPD test was done before ART initiation and after blood samples were obtained. This is the standard procedure at INCMNSZ in order to determine which group of patients to enroll in the different research projects. The PPD status is included in Table 1. We did not perform Quanti-FERON-TB Gold in-tube assay in order to check for latent TB infection because all HIV+ patients had been previously vaccinated with the BCG vaccine; moreover, the test was not comercially available in Mexico. No HIV+ patients with active pulmonary TB were included in this study. The clinical and demographic characteristics of subjects are provided in Table 1.

**Table 1:** Clinical parameters of HIV-positive patients and healthy controls

	HIV *N* (20)	HC *N* (20)	*p*
Age (years), median (IQR)	34 (19–51)	36 (23–48)	NS
Male [*n* (%)]	1 (5)	2 (10)	NS
Number of subjects	20	20	NS
Plasma HIV RNA, median (IQR), copies/ml	108,698 (4892–1.4E6)	–	–
CD4+ T cell count, median (IQR), cells/ml	242 (85–495)	–	–
PPD status [*n* (%)]	6 (30)	18 (90)	<0.05
BMI, median (IQR)	23 (18–25)	24 (21–25)	NS

*p<0.05. HC, healthy control; BMI, body mass index; IQR, interquartile range; NS, not significant.

### Ethics statement

Written informed consent was obtained from all patients and control subjects. The institutional review boards at INER and INCMNSZ approved this protocol (C29-10).

### Cells

Peripheral blood mononuclear cells (PBMCs) and plasma samples were isolated from venous blood in BD vacutainer CPT tubes (Becton Dickinson, San Jose, CA). Plasma was recovered and frozen for future cytokine analysis. PBMCs were collected and counted to determine their viability by using the trypan blue dye exclusion method.

### Monocyte enrichment

Monocytes were enriched by using positive selection via magnetic microbeads coated with antibodies to CD14 (Miltenyi Biotech, Bergisch Gladbach, Germany). The purified cells were routinely 90 to 95% of the intended cell type.

### T cell enrichment

Total T cells were enriched using negative selection via magnetic microbeads coated with antibodies to CD14, CD15, CD16, CD19, CD34, CD36, CD56, CD123 and CD235a (Pan T Cell Isolation Kit, Miltenyi Biotech). The purified cells were routinely 93 to 97% of the intended cell type.

### Cell differentiation

CD14 monocytes were plated at 1×10^5^ cells per well in 96-well plates (Costar, ON, Canada) with supplemented RPMI 1640 medium (Gibco BRL, Grand Island, NY, USA). After a seven-day incubation period, viable cells were considered to be monocyte-derived macrophages (MDMs) based on their expression profile of CD14, CD68 and the mannose receptor.

### Multiparametric flow cytometry

PBMCs were stained for 20 min at 4°C with mAb to CD3, CD4, CD8, CD14, CD16, CD38, CD56, HLA-DR, PD-1, Tim-3 (BioLegend, San Diego, CA) and Gal9 (Woburn, MA). Multiparametric flow cytometry was performed using a FACS Aria II flow cytometer (Becton Dickinson, San Jose, CA) and compensated with single fluorochrome. Data were analyzed with FlowJo software (Tree Star, San Carlos, CA). The cells used for FMO conditions were stained and acquired in parallel. Dead cells were omitted by the side scatter/forward scatter gating strategy, and isotype-matched control antibodies were used to identify background levels of staining. Typically, 100,000 events were recorded.

### Bacteria

During mid-log growth, the culture was supplemented with glycerol (6%, vol/vol), aliquoted and stored at −70°C until further use. Bacterial titres were determined by plating serial 10-fold dilutions on 7H10 agar and counting the colony-forming units (CFU) after incubation for two to three weeks. The viability of M.tb-H37Rv is retained for at least two years. At the time of use, bacteria were thawed, sonicated for 40 s by means of an ultrasonic cup horn water bath (90 W; 20 kHz; Heat Systems- Ultrasonics, Farmingdale, NY) and added to cultured macrophages as indicated.

### *In vitro* infections and co-cultures

MDM were infected with M.tb-H37Rv at a multiplicity of infection of 10 [11]. Briefly, bacteria were opsonized for 5 min using RPMI 1640 medium supplemented with 2% inactivated human serum. Bacteria were counted in a Petroff-Hausser chamber and added to MDM. Duration of infection was 2 h. At Days 1 and 4 postinfection, cells were lysed with 0.1% saponin solution, and bacterial colonies were counted after 21 days by plating five serial dilutions of cell lysates on Middlebrook 7H10 agar plates. Autologous T cells (1×10^5^/well) were added to infected MDM to achieve a final ratio of 1:1 (effector:target cell). Day 1 (d1) represents the CFU in infected MDM alone 24 h postinfection, whereas Day 4 (d4) is the CFU recovered four days postinfection in the absence of any treatment. In this study, we had only two to three agar plates where no bacteria was observed in the last serial dilution. This was an important finding in our experimental model but we cannot guarantee that bacteria died as a result of the blockade of the Tim-3-Gal9 pathway. Supernatant was removed before cell lysis; however, it is possible that some macrophages were dead at d4 postinfection. To avoid over-interpretation, we evaluated MDM viability in all our experiments. When macrophage viability was below 85% we stopped the experiment.

### Blocking assays

#### MDM-blocking

M.tb-infected MDM were incubated for 20 min with anti-human Gal9 (clone 9M1-3 BioLegend, 10 µg/ml) or isotype control antibodies. After incubation, MDM were co-cultured with autologous T cells.

#### T cell-blocking

Autologous T cells were also incubated for 20 min with anti-human Tim-3 (clone 4A4, 10 µg/ml) or the combination of anti-Tim-3/anti-PD-1 mABs. T cells were co-cultured with M.tb-infected MDM (ratio 1:1). Anti-human Tim-3 (clone 4A4) was kindly donated by Dr. Vijay Kuchroo (Brigham and Women's Hospital, Boston, MA).

### IL-1**β** treatment

To test control of M.tb growth by IL-1β in the MDM of HIV patients, 10 ng/ml of IL-1β were added directly to the media containing the infected MDM.

### Cytokine measurement by multiplex bead array kit

Supernatants were analyzed with a multiplex cytokine detection system (human Th1/Th2 cytokine Cytometric Bead Array II kit, BD Biosciences Pharmingen, San Diego, CA) and analyzed by flow cytometry (FACSDiva, BD^®^).

### Statistical analysis

Data are shown as mean±standard deviation (SD) or median interquartile range (IQR). An unpaired student's test or Mann-Whitney *U* test was used to compare two groups, and a Kruskal-Wallis test with Dunnett's post-test was used when more than two groups were compared. *p*<0.05 were considered statistically significant (GraphPad Software, Inc., San Diego, CA).

## Results

### Tim-3 and PD-1 expression on CD4+ and CD8+ T cells

**Figure 1:**
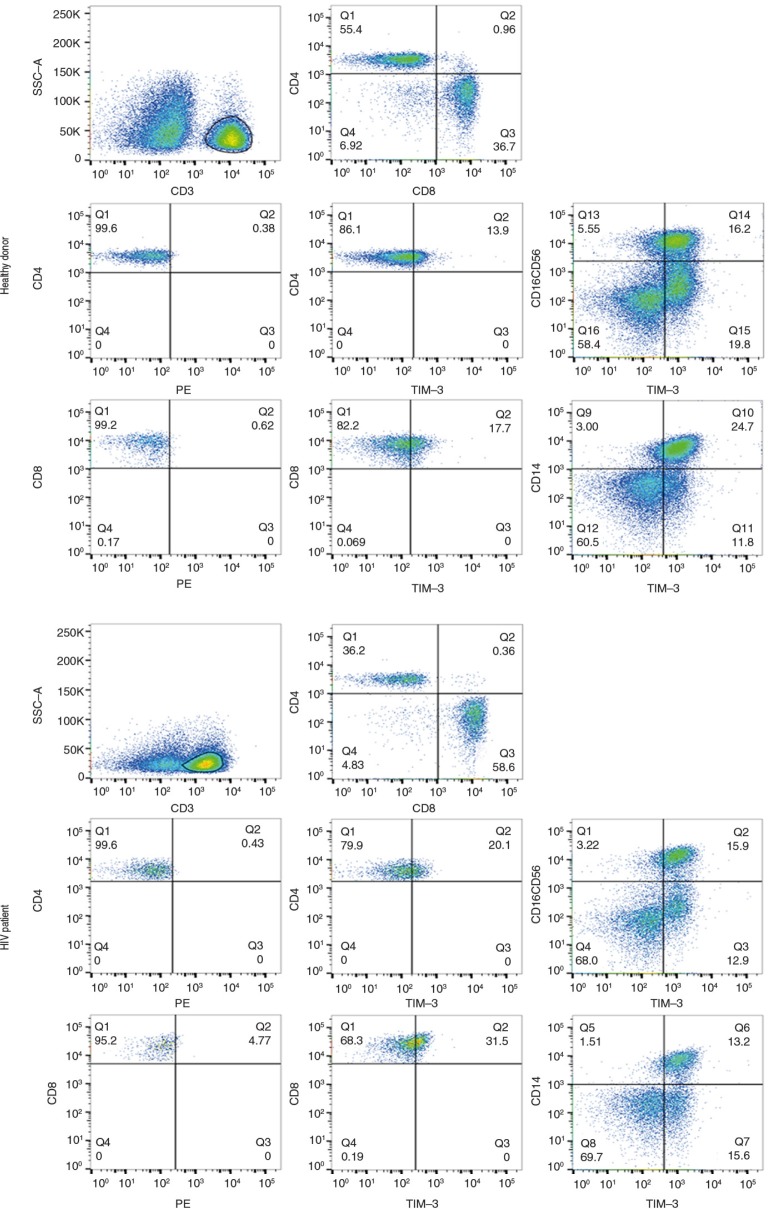
Identification of peripheral blood mononuclear cells (PBMC) expressing Tim-3. PBMC from HIV+ patients and healthy controls were stained and analyzed for cell surface markers to further delineate Tim-3 and Gal9 expression. As shown in the top panels, lymphocytes were CD3 scatter-gated and then CD4+ and CD8+ cells were gated. The remaining panels show the Tim-3 expression on CD4+, CD8+, CD14+ and NK subsets. The figure demonstrates the FMO panels for CD4+ and CD8+ T cells. Representative plots are shown. Numbers indicate the percentage of positive cells in each gate. Data were collected on a FACS Aria II flow cytometer (10 colours and 2 scatter measurements). Data were analyzed using FlowJo software.

**Figure 2:**
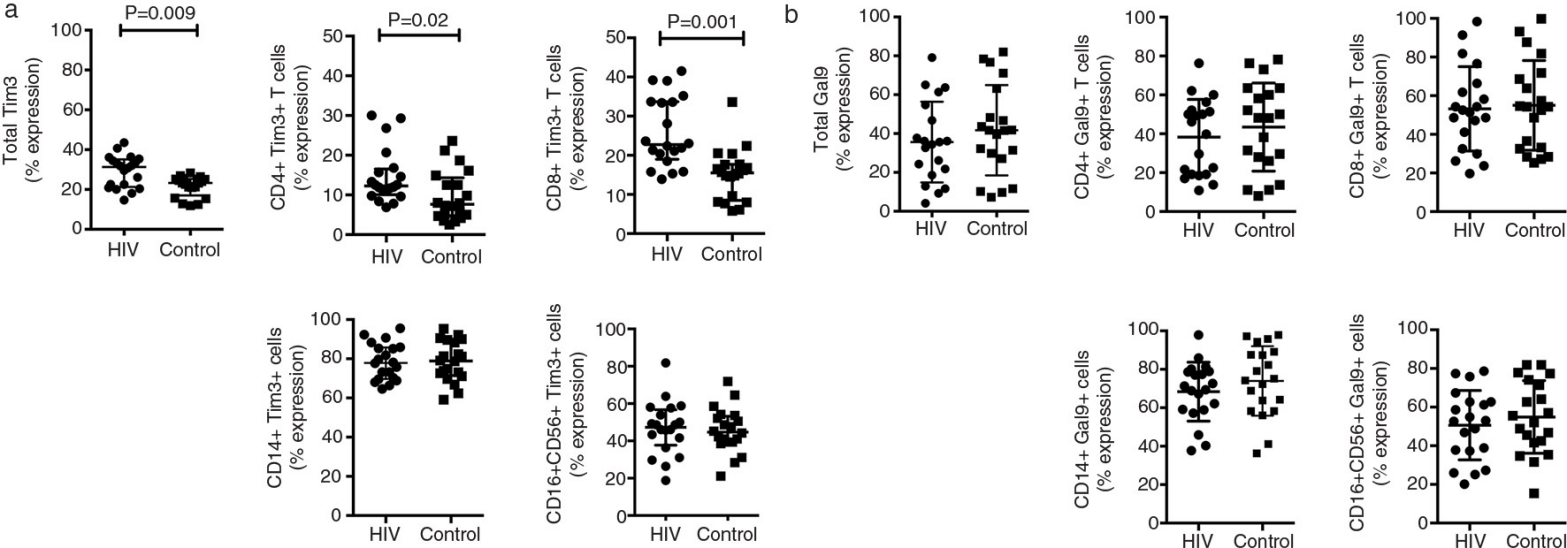
Expression of Tim-3 and Gal-9 on immune cell subsets. The frequency of Tim-3+ cells (a) and Gal-9 (b) in HIV+ patients and healthy controls was analyzed as indicated. Bars show median values and interquartile range. Statistical analyses were performed using the Mann-Whitney *U* test (*n*=20).

To analyze the frequency of Tim-3- and Gal9-positive cells, 20 naïve-to-treatment HIV+ patients and 20 healthy controls were enrolled. A higher frequency of CD3 + CD4 + Tim-3+ T cells [median 12.2 (10 to 16.5%) vs. 7.6 (4.7 to 14.3%)] (*p*=0.02) and CD3 + CD8 + Tim-3+ T cells [median 22.7 (18.9 to 33.6%) vs. 15.5 (8.4 to 17.6%), respectively] (*p*=0.001) was detected in the HIV+ patients compared to control group (Figures 1 and 2a). The frequency of monocytes expressing Tim-3 and Gal9 in HIV+ patients was not different from that in healthy controls [median 70.3 (58.9 to 78 to 8%) vs. 74.6 (59.6 to 88.7%) and median 31.3 (21.3 to 35.1%) vs. 23.2 (17.0 to 25.2%) respectively]. Similar findings were identified when Tim-3 was analyzed on NK cells [median 47.3 (37.7 to 56.6%) vs. 44.7 (39.4 to 53.2%)].

**Figure 3:**
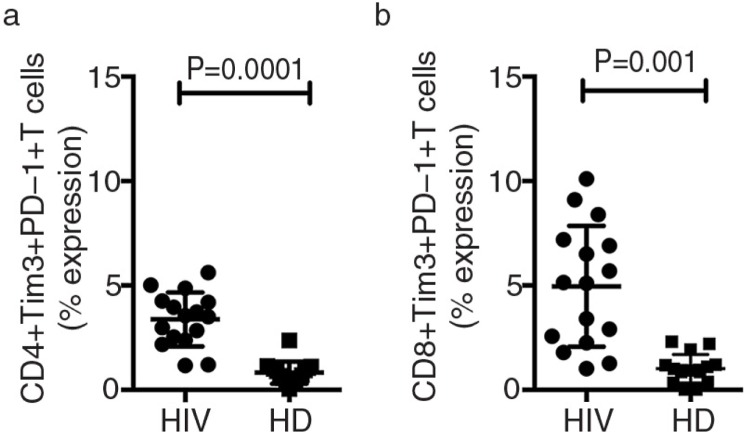
Frequency of CD4 + Tim-3 + PD-1+ and CD8 + Tim-3 +PD-1+ T cells (a and b). The frequency of double-positive cells in HIV+ patients and healthy controls were analyzed as indicated. Bars show median values and interquartile range. Statistical analyses were performed using the Mann-Whitney *U* test (*n*=16).

**Figure 4:**
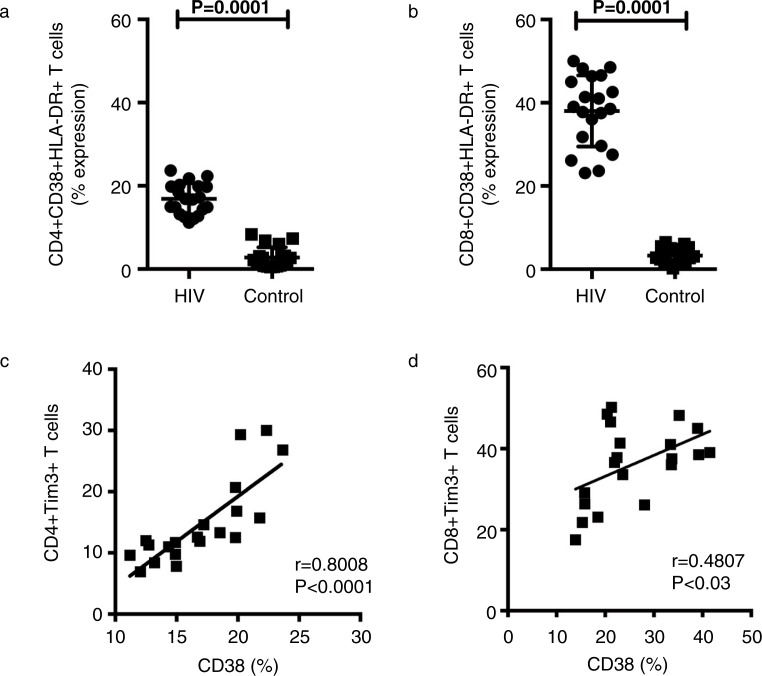
Tim-3 is up-regulated on CD4 and CD8 T cells in HIV+ patients, and its expression correlates with CD38 expression. (a, b) The percentages of CD38+ cells within CD8+and CD4+T cell populations are indicated for 20 HIV+ patients and healthy controls. Statistical analyses were performed using the Mann-Whitney *U* test. (c, d) Correlations between Tim-3 expression on CD8+ and CD4+ T cells and levels of CD38 expression among HIV+ patients and healthy controls are shown. Statistical analyses were performed using the Spearman's rank correlation test.

Expression of Gal9 in HIV+ patients and controls showed no differences (Figure 2b). PD-1 expression on T cells increased during chronic HIV infection; we found that basal expression of PD-1 and Tim-3 increased in HIV+ patients compared with the control group (Figure 3a, b) (*p*=0.001). T cell activation was analyzed by CD38 and HLA-DR expression. The higher frequency of CD4 + CD38+ and CD8 + CD38+ T cells correlated with the level of expression of Tim-3 (*p*<0.0001 and *p < *0.03) (Figure 4a, b, c, d).

### Control of bacterial growth

**Figure 5:**
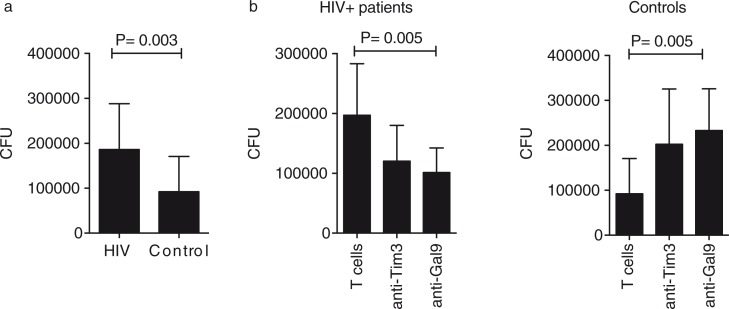
Tim-3–Gal9 interaction participates in the immune response against *Mycobacterium tuberculosis* (M.tb). (a) M.tb-infected monocyte-derived macrophages were cultured with T cells alone or in the presence of 10 mg/ml of anti-human Tim-3 or anti-human Gal9 mAb (b). Colony-forming units were recovered on Day 4 postinfection. Horizontal bars represent median values and interquartile range. Kruskal-Wallis test compared with T cells. *n*=20.

We evaluated the ability of MDM and T cells from HIV+ patients to restrict bacterial growth in the absence of any treatment, and we determined that bacterial replication was inhibited by 49.8%±1.5 in HIV+ patients and 72.3%±21.7 in the control group (*p*=0.0007) (Figure 5a). The level of suppression of bacterial replication in HIV+ patients without ART has not been analyzed previously, and these results demonstrate that even when monocytes have been chronically exposed/infected to HIV, they conserve some mechanisms that are important for controlling bacterial growth.

### Controlling bacterial replication by blocking Tim-3 and PD-1

**Figure 6:**
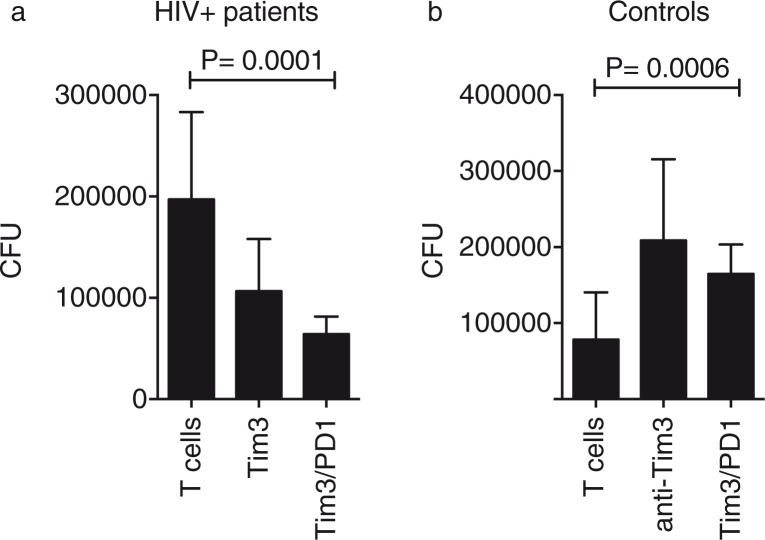
Blocking of Tim-3 and PD-1 inhibitory receptors restore macrophage and T cell control of bacterial growth (a). *Mycobacterium tuberculosis*-infected monocyte-derived macrophages were cultured with T cells alone or in the presence of 10 mg/ml of anti-human Tim-3 or anti-human PD-1 mAb (b). Colony-forming units were recovered on Day 4 postinfection. Horizontal bars represent median values and interquartile range. Kruskal-Wallis test compared with T cells. *n*=20.

We had previously demonstrated that blocking Tim-3/Gal9 interaction increased bacterial growth when compared to conditions in which macrophages were cultured without blocking antibodies. That finding was the result of the limited pro-inflammatory cytokine production [12]. Given that Tim-3 expression has been associated with a T cell-exhausted phenotype, we hypothesized that by blocking Tim-3 in HIV+ patients we could improve the ability of macrophages and T cells to restrict bacterial replication. In effect, blocking Tim-3 expression on T cells from HIV+ patients decreased the CFU compared to the untreated experimental conditions (127.8%±62.2 vs. 49.8%±1.5, respectively). We next analyzed whether by blocking Gal9 on MDM we could get a similar result. Upon Gal9 blocking, we observed restriction of bacterial growth; however, it was less dramatic than the result observed with Tim-3 (Figure 5b). In contrast, when the control group was analysed, more CFU were counted in the condition in which blocking antibodies were added [12]. To determine whether the double-blocking of Tim-3 and PD-1 increased control of bacterial growth, T cells were treated with anti-Tim-3 plus anti-PD-1 blocking antibodies. We identified that double-blocking did not lead to a more control of bacterial growth when compared with Tim-3 blocking alone (Figure 6a). Once again, we observed the opposite phenomenon with the control group (Figure 6b). These data demonstrated that Tim-3 and PD-1 are two molecules that when expressed together can negatively regulate T cells. However, they also suggest that in order to get a functional immune response there must be a fine balance in their expression profile.

### The antimicrobial effect of Tim-3 and PD-1 blocking requires functional T cells

**Figure 7:**
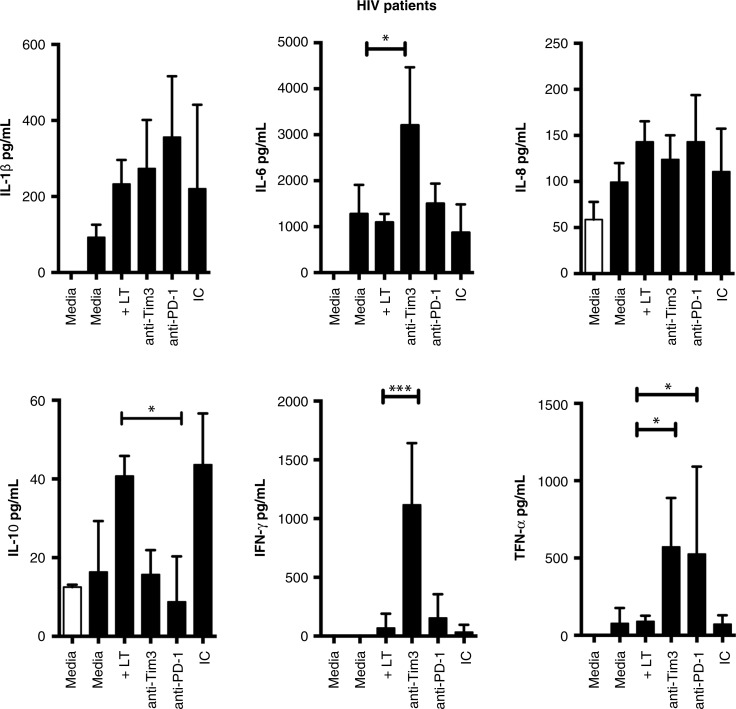
Blocking of Tim-3 and PD-1 inhibitory receptors increases pro-inflamatory cytokine production in HIV+ patients. Pro-inflammatory and anti-inflammatory cytokines were measured in culture supernatants from HIV+ patients (*n*=20). Bars represent median values and interquartile range. Kruskal-Wallis test compared with T cells.

To ascertain whether the effect of the double-blocking of Tim-3 and PD-1 pathways was related to a specific cytokine profile, we measured a panel of cytokines in the culture supernatant from the earlier experiments. This analysis showed that *in vitro* blocking of Tim-3 and PD-1 synergistically restores T cell production of IL-6, IFN-γ and TNF-α (Figure 7). More interesting was the finding that in the absence of the blocking antibodies IL-10 was produced, suggesting that total T cells may induce a regulatory phenotype in immune dysfunction during HIV infection. However, these experiments did not provide conclusive evidence as to whether the Tim-3 and PD-1 pathways had overlapping functions.

### Tim-3 expression over time and its impact on bacterial growth

**Figure 8:**
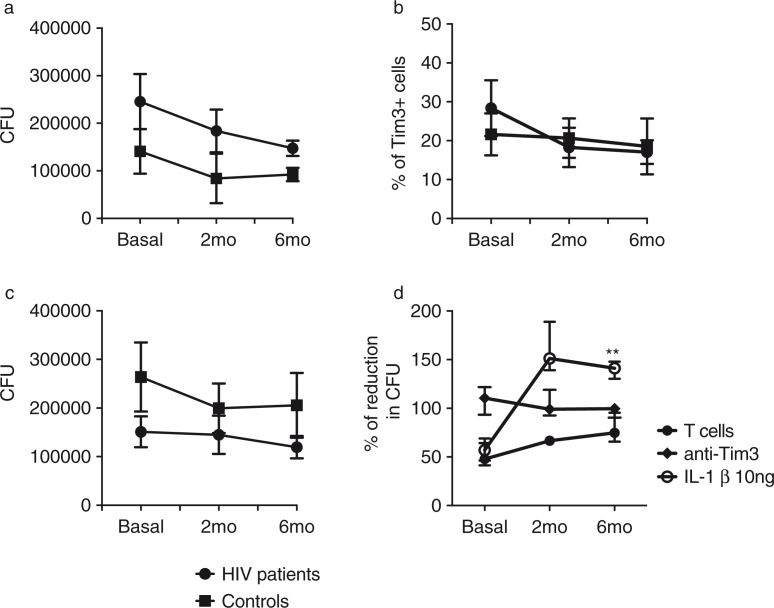
Effect of anti-retroviral therapy (ART) on the immune response against *Mycobacterium tuberculosis* (M.tb) during HIV infection. HIV+ patients (*n*=10) and healthy controls (*n*=10) were sampled at baseline; eight HIV+ patients and ten healthy controls at 2; and seven HIV+ patients and ten healthy controls at 6 months after initiation of ART. (a) M.tb-infected monocyte-derived macrophages (MDM) were cultured with T cells to evaluate their ability to restrict bacterial growth; (b) basal Tim-3 expression on total T cells vs. 2 and 6 months after initiation of ART; (c) M.tb-infected MDM were cultured with T cells and temporal analysis of bacterial growth under the presence of anti-Tim-3 and anti-Gal9 antibodies was analyzed; and (d) IL-1**β** restricts intracellular bacterial replication in M.tb-infected MDM more efficiently than Tim-3 blocking. Data are presented as median values and interquartile range. Kruskal-Wallis test compared colony-forming units at months among treatments.

To identify whether the initiation of ART in HIV+ patients increased the functionality of macrophages and T cells in a Tim-3-dependent manner, we repeated our *in vitro* experimental model at two and six months after initiating ART. Six months of therapy were sufficient to increase T cell capacity to control bacterial growth to the same level as the control group. Figure 8a shows the number of CFU in the absence of blocking antibodies. This result demonstrates that after ART the functionality of macrophages and T cells increased over time to a similar level to that observed in healthy donors. These results suggest that ART improves the antibacterial defence mechanisms critical for HIV+ patients, especially those who live in countries where pulmonary TB is endemic. We also found that six months of treatment were sufficient to reduce the frequency of total Tim-3+ cells to the control group level (Figure 8b). When we analyzed bacterial growth at the three time points in the presence of blocking antibodies we observed that HIV+ patients had a better control than the control group (Figure 8c). Next, we compared the effectiveness of Tim-3 blocking with Il-1β macrophage treatment, which we had demonstrated as an effective way of restricting bacterial growth [10]. We found that, while Tim-3 blocking did not change over time, with IL-1β almost no CFU were observed in the agar plate, suggesting that the bacteria were killed (Figure 8d).

**Figure 9:**
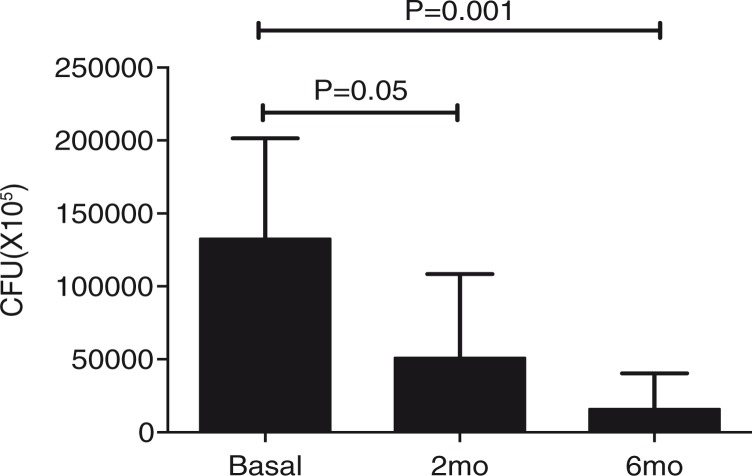
Temporal analysis of bacterial growth in monocyte-derived macrophages (MDM) infected by *Mycobacterium tuberculosis* (M.tb). M.tb-infected MDM were colony-forming units from M.tb-infected MDM were recovered on Day 4 postinfection. This analysis was done on three consecutive time points (basal, 2 and 3 months after initiation of anti-retroviral therapy). Horizontal bars represent median values and interquartile range. Kruskal-Wallis test compared with basal bacterial growth. *n*=10.

Finally, to verify that ART-administration-improved control of bacterial growth was dependent on both T cells and MDM, we did a temporal analysis of the CFU from M.tb-infected MDM alone and we identified that after six months of ART the number of colonies was decreased even when T cells were not present (Figure 9). This is a well-known benefit resulting from the patient's increased ability to control opportunistic infections.

## Discussion

We identified several important findings between HIV+ patients and healthy donors after a six-month follow-up period. We confirmed that expression of Tim-3 and PD-1 was higher in HIV+ patients. When analyzing Gal9 expression we did not identify significant differences, but we observed a broad distribution of Gal9 expression between groups. We consider that this result might be a consequence of the many roles that galectins play in the immune system. Next, we evaluated the activation status of T cells by analyzing CD38 and HLA-DR expression. The frequency of activated CD4+ and CD8+ T cells correlated with the frequency of CD4 + Tim-3+ and CD8 + Tim-3+ T cells. Other authors have described this phenotype [13]; however, we had to confirm these characteristics before analyzing the magnitude of the immune response against M.tb. Our first result was that MDM and T cells from HIV+ patients that had not received ART were able to markedly reduce bacterial growth, not to the same level as the control group, but close to 50%, which is very significant for these patients with chronic immunodeficiency. Many interesting questions arise from this finding, but one that is very appealing is to determine whether this response is secondary to the presence of M.tb antigen-specific memory T cells. To analyze whether Tim-3 plays a role in the immune response against M.tb in HIV, we used specific monoclonal antibodies. When Tim-3 was blocked in T cells cultured with M.tb-infected MDM from HIV+ patients, we found that the capacity to limit bacterial growth increased almost to the level that in some experiments no CFU were identified. A similar, but less dramatic, result was obtained when Gal9 expressed by MDM was blocked. Together, these results suggest that although Tim-3 expression was not very high in our cohort of HIV+ patients blocking this molecule had a great impact on immune response against M.tb. We have published our finding that blocking the Tim-3 pathway in healthy donors had a deleterious effect against M.tb, and this study ratifies that result. We believe that Tim-3 must have a highly balanced expression on T cells in order to regulate T cell activation or other immune mechanisms that mediate control of bacterial growth. PD-1 is highly expressed by virus-specific CD8 T cells during HIV infection [14, 15]. Many groups have demonstrated that blocking the PD-1 pathway leads to increased T cell proliferation and effector cytokine production [16, 17]. Based on that evidence we blocked Tim-3 alone or both Tim-3 and PD-1. We found that double-blocking did not enhance the control of bacterial growth measured with Tim-3 blocking alone. This result suggests that, at least in this particular experimental model, PD-1 did not play a critical role. Based on the results of the cytokine analysis we proposed that Tim-3 played the most critical role in the immune response against M.tb. We found that in the presence of Tim-3 blocking antibodies, the production of IL-6, IFN-γ and TNF-α increased, but IL-10 decreased. This profile is very useful in inducing macrophage activation and restricting bacterial growth. With the PD-1 blocking antibody we did not detect strong IL-6 and IFN-γ production, but high TNF-α and reduced IL-10. Collectively, this evidence suggested that Tim-3 does indeed play a more important role than PD-1, at least under our experimental conditions. Previous studies in HIV+ patients have shown that by blocking the Tim-3 and PD-1 pathways T cell proliferation capacity and effector function may be restored, but this is the first scientific evidence that blocking these molecules improves the ability to control bacterial growth against one of the most important death-causing pathogens in HIV+ patients. We do not know whether the benefit of the single- and double-blocking experiments is a consequence of longer T cell survival or of the cytokine profile that facilitates control of bacterial growth; therefore, more mechanistic studies are required to define these issues.

Finally, we analyzed the effect of ART administration on the ability of MDM and T cells to control M.tb growth. It was interesting to see that after six months of treatment immune cells from HIV+ patients reached almost the same level as healthy controls in terms of restricting bacterial growth. This is another novel result. While many physiological benefits have been described for ART, there are to date no descriptions of its benefit in *in vitro* experimental systems related to the control of M.tb growth. We believe that this is another important argument to advocate ART administration in countries where M.tb infection is endemic. The improvement that our HIV+ patients showed was not related to significant changes in the level of expression of Tim-3, as shown in Figure 7b. Finally, we also demonstrated that IL-1β administration to M.tb-infected MDM was sufficient to restrict bacterial growth in healthy donors, so we repeated the same strategy in M.tb-infected MDM from HIV+ patients and found that, indeed, IL-1β treatment proved to be much more effective than Tim-3 blocking. While this may be secondary to inflammasome activation and cytokine secretion, this study was not meant to demonstrate that specific mechanism. Our study has some limitations. We did not address latent TB infection in HIV+ patients through the IFN-γ release assay in response to the RD1-antigens ESAT-6 and CFP-10 because at the time patients were enrolled in the study this type of assay was not available at INER. Additionally, it is very challenging to properly distinguish latency among immune-compromised patients that have been exposed to BCG during childhood and to environmental mycobacteria in developing countries where pulmonary TB is an endemic disease. Instead, all subjects included in the study had the tuberculin skin test and a chest x-ray to exclude active TB. First, we used a sample size of 10 for the Gal9 and PD-1 blocking experiments due to the high number of cells that are required for such experiments. Hence, it would be important to include all experimental conditions with all patients using the same blood samples. Second, only seven HIV+ patients attended the third appointment (at six months). Third, we did not probe some important mechanisms that underlie these critical pathways, again because of the high number of cells needed for the *in vitro* infection model.

## Conclusions

Our study demonstrates, for the first time, that Tim-3 directly participates in the adaptive immune responses against M.tb infection in HIV+ patients. Blocking Tim-3 and PD-1 molecules may potentially offer a novel strategy for controlling M.tb growth, especially in countries where HIV and TB are highly prevalent. ART increases the ability of T cells and macrophages *per se* to control M.tb growth.
